# Identifying genes targeted by disease-associated non-coding SNPs with a protein knowledge graph

**DOI:** 10.1371/journal.pone.0271395

**Published:** 2022-07-13

**Authors:** Wytze J. Vlietstra, Rein Vos, Erik M. van Mulligen, Guido W. Jenster, Jan A. Kors

**Affiliations:** 1 Department of Medical Informatics, Erasmus MC, University Medical Center Rotterdam, Rotterdam, the Netherlands; 2 Data Science, Life Science Operations Department, Elsevier B.V., Amsterdam, the Netherlands; 3 Department of Methodology & Statistics, Maastricht University, Maastricht, the Netherlands; 4 Department of Urology, Erasmus MC, University Medical Center Rotterdam, Rotterdam, the Netherlands; QIMR Berghofer Medical Research Institute, AUSTRALIA

## Abstract

Genome-wide association studies (GWAS) have identified many single nucleotide polymorphisms (SNPs) that play important roles in the genetic heritability of traits and diseases. With most of these SNPs located on the non-coding part of the genome, it is currently assumed that these SNPs influence the expression of nearby genes on the genome. However, identifying which genes are targeted by these disease-associated SNPs remains challenging. In the past, protein knowledge graphs have often been used to identify genes that are associated with disease, also referred to as “disease genes”. Here, we explore whether protein knowledge graphs can be used to identify genes that are targeted by disease-associated non-coding SNPs by testing and comparing the performance of six existing methods for a protein knowledge graph, four of which were developed for disease gene identification. We compare our performance against two baselines: (1) an existing state-of-the-art method that is based on guilt-by-association, and (2) the leading assumption that SNPs target the nearest gene on the genome. We test these methods with four reference sets, three of which were obtained by different means. Furthermore, we combine methods to investigate whether their combination improves performance. We find that protein knowledge graphs that include predicate information perform comparable to the current state of the art, achieving an area under the receiver operating characteristic curve (AUC) of 79.6% on average across all four reference sets. Protein knowledge graphs that lack predicate information perform comparable to our other baseline (genetic distance) which achieved an AUC of 75.7% across all four reference sets. Combining multiple methods improved performance to 84.9% AUC. We conclude that methods for a protein knowledge graph can be used to identify which genes are targeted by disease-associated non-coding SNPs.

## 1 Introduction

A common way to identify which genetic variations are associated with a disease is by comparing the genetic code of a healthy population to that of a patient population in genome-wide association studies (GWAS). GWAS have identified many single nucleotide polymorphisms (SNPs) that play important roles in the genetic heritability of traits and diseases such as prostate cancer [1]. However, because the majority of these SNPs are located on the non-coding part of the genetic code (more than 90% of the SNPs for prostate cancer [2]), it is not yet clear how they increase the risk of disease. These SNPs do not directly affect the protein coding sequence as non-synonymous SNPs in the protein coding sequence do, or intronic SNPs that may change splicing, but it is currently assumed that a non-coding SNP influences the expression of the nearest gene on the genome [3]. However, a non-coding SNP does not always correlate with the differential expression of the nearest gene. This phenomenon is illustrated by the obesity-associated SNP rs9930506, which is found in an intron on the *FTO* gene, but targets the *IRX3* gene, which is nearly 487 kilobases (kb) away [4]. How these non-coding SNPs contribute to their associated phenotype is investigated in post-GWAS analyses [5]. An important first step in such an analysis is to identify which gene is targeted by the non-coding SNP. Existing tools such as FUMA [6], VEGAS [7], MAGENTA [8], Pascal [9], DEPICT [10], and more recently OpenTargets [11] leverage genomic position, aggregated GWAS data, existing eQTL data, 3D chromatin interactions, or functional annotations to identify the genes targeted by SNPs.

In another field, referred to as disease gene identification, methods have been developed to computationally identify genes that are associated with diseases [12]. What constitutes a “disease gene” is loosely defined, and can both encompass genes which have a protein-changing mutation [13], as well as genes that are differentially expressed between healthy and diseased subjects [5]. Unfortunately, differentially expressed disease genes often do not differentiate between genes whose expression is changed by a SNP, downstream effects of other genes, or other factors such as epigenetic modifications. Nonetheless disease gene identification is a task that is closely related to identifying which genes are targeted by disease-associated SNPs, with methods that may be applicable to both tasks.

Many disease gene identification methods are based on knowledge graphs that only consist of proteins, commonly referred to as protein-protein interaction networks. Through the involvement of proteins in metabolic, signaling, immune, and gene-regulatory networks, protein knowledge graphs can help to mechanistically explain disease and physiological processes [14]. Because proteins are encoded by genes, genetic variations can affect the way proteins interact with each other, thereby disrupting molecular pathways and ultimately leading to disease. Besides using protein knowledge graphs for disease gene identification, they have been commonly used for many types of analyses [15], such as drug efficacy screening [16, 17] identifying interrelationships between diseases [18, 19], and drug target identification [20]. Within protein knowledge graphs, biomedical knowledge published in literature and databases is formalized as subject–predicate–object triples, where pairs of entities, such as proteins, are related to each other by verbs or predicates [21, 22] An example of such a subject–predicate–object triple is *MAPK*–regulates–*ALOX5*.

Here, we explore whether protein knowledge graphs can be used to identify genes that are targeted by disease-associated SNPs which are located on the non-coding part of the genome. We test four previously published methods for disease gene identification. Predicate information is not used by existing disease-gene identification methods, but has been shown to improve performance in analyses performed on protein knowledge graphs [17]. We therefore also test two existing methods that utilize the predicates in our knowledge graph. The performance of the selected methods is compared against two baselines: one is based on the leading assumption that SNPs target the nearest gene on the genome, the other is DEPICT, a state-of-the-art and commonly used method that is based on gene annotations and uses guilt-by-association [10].

We test our selected methods on two diseases. The first disease is prostate cancer, which is estimated to be the second most common type of cancer in the world for men [23], and with an estimated heritability of 57%, is one of the cancers that is most influenced by genetics [24]. The second disease is coronary artery disease, the leading cause of death worldwide [25], where a strong genetic effect on death especially at younger age has been found [26].

## 2 Methods

### 2.1 Knowledge graph

We based our experiments on Resnet, the graph database underlying Elsevier’s Pathway Studio product [27, 28]. Within this knowledge graph, the nodes represent biomedical entities such as proteins, diseases, physiological processes, and drugs, while the edges between the nodes describe their interrelationships, e.g. regulates, expresses, modifies. The types of interrelationships that can exist between nodes have been defined by experts and form the predicates in triples. Predicates, together with the entities, were computationally extracted from PubMed and 43 publicly available full-text journals [27]. Information about the articles from which predicates were extracted is linked to the edges as provenance. Resnet does not distinguish between a gene and the protein for which that gene encodes, and maps both to the same node. For our research, we only used protein information in Resnet, excluding all information about non-protein-coding sequences and mitochondrial genes.

### 2.2 Reference sets

Reference sets of SNP-gene pairs were obtained from (1) a study that performed a literature review of SNP-gene pairs [3], (2) an expression quantitative trait loci (eQTL) study [29], and (3) a meta-analysis of GWAS [30]. A fourth reference set was created by selecting a high-confidence subset from the results of the literature review.

In all reference sets, deprecated SNP identifiers and gene symbols were manually updated to match dbSNP (https://www.ncbi.nlm.nih.gov/snp) for SNPs, and the NCBI gene catalogue (https://www.ncbi.nlm.nih.gov/gene) for gene symbols. Genomic locations of genes and of SNPs were retrieved with the Python packages PyEnsembl (using ENSEMBL release 92) and myvariant, respectively.

Because the disease gene identification methods that were tested have been developed for protein-protein interaction networks, we excluded all non-protein-coding sequences from our reference sets. Furthermore, because our objective is to identify genes targeted by non-coding SNPs, all entries containing SNPs on the coding region/exon were excluded as well. Finally, in line with previous work, we set a maximum distance of 2000 kb between SNPs and gene candidates [10, 31]. For each reference set, the number of SNP-gene pairs that did not fulfil these criteria are shown in S1 Table.

In addition to the maximum distance of 2000 kb between SNPs and genes, we also tested smaller maximum distances, i.e. 25, 50, 100, 500, 1000 kb. Furthermore, beside these static distances, we also tested the maximum distance based on linkage disequilibrium as determined by DEPICT [10].

To align with common terminology in the machine learning field we refer to SNP-gene pairs found in the reference sets as positive cases, while negative cases are SNP-gene pairs consisting of all genes that are (partially) within the selected range of a SNP in the reference set, but are not mentioned as the target of the SNP. It is possible for SNPs to only have positive cases within the interval. It is also possible for genes to be located within the interval of multiple SNPs, or for one SNP to target multiple genes. In both situations, each SNP-gene pair is counted as a separate case. For each reference set, the number of cases for different maximum genetic distances is shown in Table 1.

**Table 1:** Number of positive and negative cases in the reference sets for different genetic intervals. Maximum genetic distance indicates the maximum allowed distance between a SNP and a gene. A variable maximum distance is based on the linkage disequilibrium as determined by DEPICT. Number of SNPs is the number of SNPs for which at least 1 positive case was found within the genetic interval.

Reference set	Maximum genetic distance	No. of SNPs	Total no. of positive cases	Total no. of negative cases	Median no. of negative cases per SNP
Farashi	25 kb	32	33	28	1
	50 kb	47	48	73	2
	100 kb	58	59	174	2
	500 kb	143	148	2266	11
	1000 kb	188	196	5422	19
	2000 kb	213	225	10,863	33
	Variable	97	97	211	3
Farashi high-confidence	25 kb	16	17	22	2
	50 kb	21	22	36	2
	100 kb	24	25	83	2
	500 kb	62	66	1125	12
	1000 kb	84	90	2914	20
	2000 kb	95	101	5678	38
	Variable	46	46	85	2
DeRycke	25 kb	16	20	19	1,5
	50 kb	23	29	33	1,5
	100 kb	30	37	84	2
	500 kb	78	126	1160	10,5
	1000 kb	97	168	2580	17
	2000 kb	109	191	5392	36
	Variable	45	49	113	2
Teslovich	25 kb	8	8	13	1,5
	50 kb	15	15	36	2
	100 kb	23	23	88	4
	500 kb	65	68	878	9,5
	1000 kb	81	84	1998	17
	2000 kb	94	97	4288	32
	Variable	70	71	147	2

#### 2.2.1 Farashi reference set

The first reference set is based on a comprehensive literature review of prostate-cancer associated SNPs and their gene targets performed by Farashi et al. [3]. We used the complete set as well as a high-confidence subset that only includes the entries with a p-value less than 5 × 10^−8^. Entries for which no p-values were provided in Farashi’s overview were excluded from the high-confidence set.

The review by Farashi provided 1139 distinct SNPs and 271 distinct genes, extracted from 26 distinct publications. Whereas most studies in Farashi’s review describe genes that are targeted by one or two SNPs, one study identified 5 genes that were each targeted by more than a hundred SNPs [32]. We considered this study to be an outlier and removed all its results from our reference set.

The final set consists of 225 positive cases, including 213 distinct SNPs and 191 distinct genes. Of these, 101 positive cases (95 distinct SNPs and 86 distinct genes) met the p-value criterion of the high-confidence set. Within a distance of 2000 kb from the SNPs, 10,863 negative cases were found for the complete set and 5678 for the high-confidence set. The Farashi reference set is available in S2 Table.

#### 2.2.2 DeRycke reference set

The reference set derived from the literature review by Farashi et al. was based on studies that used different experimental methods, which may act as a confounder. We therefore included another reference set for prostate cancer based on a recent, single eQTL study by DeRycke et al. [29], who compared RNA sequencing data obtained from 471 normal and 249 at risk samples. This study has been published after Farashi’s literature review, and its results are therefore not included in that set.

The set of DeRycke consists of 322 distinct SNPs and 215 distinct genes. After removing all ineligible SNP-gene pairs, the final DeRycke reference set consists of 109 distinct SNPs that target 122 distinct genes, forming 191 positive cases. Within a 2000 kb distance of the SNPs there are 5392 negative cases. The DeRycke reference set is available in S3 Table.

#### 2.2.3 Teslovich reference set

Teslovich et al. performed a meta-analysis of 46 GWAS, comprising more than 100,000 individuals in total, to identify SNPs associated with risks factors for coronary artery disease (i.e. total cholesterol, low-density lipoprotein cholesterol, high-density lipoprotein cholesterol, and triglycerides) [30]. Based on this meta-analysis they identified 102 SNPs targeting 98 genes with a p-value lower than 5 × 10^−8^, from which three were experimentally validated with mouse models. After removing all ineligible SNP-gene pairs, the final reference set consists of 94 distinct SNPs and 91 distinct genes, in 97 positive cases. Within the 2000 kb range of the SNPs there are 4288 negative cases.

This set has previously been used in the Benchmarker study by Fine et al. [33], to evaluate DEPICT [10], and to evaluate the predecessor of DIAMOnD [34]. The Teslovich reference set is available in S4 Table.

### 2.3 Experimental setup

We selected six existing protein-knowledge graph based methods listed in Table 2 (node2vec, RDF2vec, metapaths, network distance, graphlets, DIAMOnD) to generate features from the protein knowledge graph. Parameters for the feature generation methods were set to default except for node2vec (see Section 2.4.5).

**Table 2:** Overview of the various methods and experimental settings.

Methods	Variations	Classifiers	Maximum genetic distance (in kb)
Node2vec	• No modification • Autoencoding • Graphlets • Autoencoding + graphlets	• Logistic Regression • Support-Vector Machine • Decision Tree • Random Forests	• 25 • 50 • 100 • 500 • 1000 • 2000 • Variable (DEPICT)
RDF2vec			
Metapaths	• Frequency • Binary		
Network distance	Not applicable		
Graphlets	• Frequency • Log of frequency		
DIAMOnD	Not applicable		
Genetic distance	Not applicable		
DEPICT	Not applicable		

Apart from DIAMOnD and the graphlets, the disease gene identification methods tested by us were trained and evaluated using the supervised learning algorithms that were used in the original studies. These are: logistic regression (LR) (used by Agrawal et al. [35] and by Ristoski et al. [36]), support-vector machines (SVM) (used by Peng et al. [37]), decision trees (DT) (used by Ristoski et al. [36]), and random forest (RF) (used by Vlietstra et al. [19]). We excluded the K-nearest neighbour classifiers used by Xu et al. [38], Ristoski et al. [36], and Milenković et al. [39] due to their limited ability to rank, often leading to tied ranks for genes. To enable a fair comparison, every feature set was tested with each classifier. Classifier parameter settings were taken from the original work, or if not specified left on default.

Because the same gene can be a candidate for multiple SNPs that are in close proximity to each other on the genome, and their status as a positive or negative case can differ for these SNPs, performing cross-validation with randomized folds could lead to the same gene occurring in both in the training and the test set. Therefore, to determine whether the selected methods can be used to identify genes that are targeted by SNPs, we followed the leave-chromosome-out cross-validation methodology recommended by Fine et al. [33]. For every cross-validation experiment, all SNPs and their gene candidates that are located on a single chromosome are used as a test set, while all other SNPs and their gene candidates on the other chromosomes are used as the training set. Therefore, leave-chromosome-out cross-validation can consist of a maximum of 23 cross-validation experiments (24 if counting X and Y chromosomes separately). Depending on the distribution of the SNPs across the genome, these folds often have unequal sizes. Because folds are fixed in leave-chromosome-out cross validation, only a single cross-validation experiment needs to be performed.

**Fig 1:**
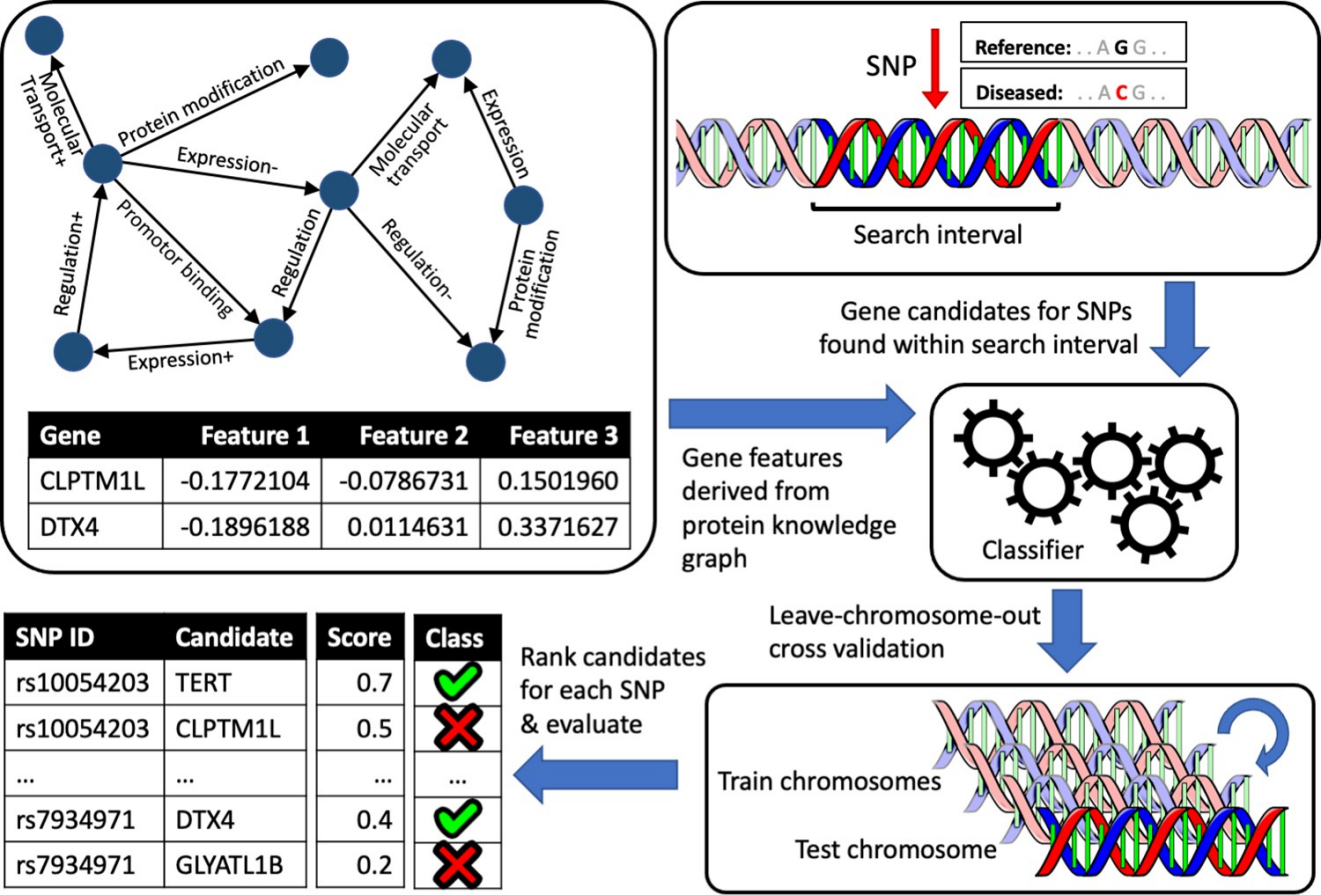
Overview of the experimental process. A feature vector is created for every gene based on the protein interactions in the knowledge graph. The figure shows features generated with node2vec. Simultaneously, the gene candidates for every SNP are identified based upon their proximity to the SNP on the genome. The size of the interval can either be pre-defined, e.g. at 50 kb, or be determined by DEPICT based upon the linkage disequilibrium of the SNP with other SNPs as found in the HapMap or 1000 genomes project data. Based upon the positive and negative cases in each reference set, a classifier is trained and evaluated using the leave-chromosome-out methodology, where all SNP-gene pairs on one chromosome are used as a test set while those on the other chromosomes are used as the training set. All candidate genes for a SNP on the test chromosome are assigned scores by the classifier, based upon which the candidates are ranked. Finally, the ranking of gene candidates for every SNP is evaluated using the reference sets.

Candidate genes within a pre-defined range of a SNP were ranked based upon the scores assigned to them by the classifier. For each SNP, performance was first measured individually with the area under the receiver operating characteristic curve (AUC) and recall of the positive cases in the top-1 and top-3 ranked genes. Subsequently, the average performance was measured across all SNPs in the reference set. An overview of the experimental setup is shown in Fig 1. For each combination of method and classifier, we present the average and the minimum and maximum values of the performance metrics across all reference sets. The performance of each method is compared to two baseline methods: one ranking the gene candidates based on their genetic distance from the SNP, the other DEPICT [10].

A complete overview of all methods, their variations, the tested genetic intervals, and machine learning classifiers can be found in Table 2. Code and data can be found in the following GitHub repository: https://github.com/wjvlietstra-els/Post-GWAS.

### 2.4 Tested methods

#### 2.4.1 DEPICT

DEPICT is a commonly used post-GWAS analysis method that is based on guilt-by-association (i.e. co-occurrence of genes within pre-defined gene sets) to identify genes and pathways that are affected by SNPs [10]. Within a genomic range of 2000 kb, DEPICT assigns the SNP with the lowest p-value as the lead SNP. The pairwise linkage disequilibrium between this SNP and all other SNPs on the chromosome is calculated based on HapMap data and the 1000 genomes data [40, 41]. The border of a locus is determined by the location of the most distant SNP that has a linkage disequilibrium with a correlation (r^2^) higher than 0.5. All genes located at least partly within this range are considered to be part of the locus, and therefore candidates for all SNPs that are found within it. Loci with overlapping genes are merged. When a locus does not contain any genes, DEPICT selects the nearest gene.

DEPICT prioritizes gene candidates based on their correlation with genes that are found in other loci with which they share membership of pre-defined gene sets. DEPICT contains 14,461 of these gene sets that are defined by the shared annotations between genes in GO, KEGG, REACTOME, neighbouring genes in the InWeb protein-protein interaction network [42], and shared phenotypes as found in the Mouse Genetics Initiative [43]. Only sets consisting of at least 10 and at most 500 genes are extracted from these databases. These sets are expanded by genes that are often co-regulated with the genes in these sets, as calculated with almost 78,000 microarray datasets of human, mouse, and rat gene expression data from the GEO database. Memberships of genes to a specific gene set are indicated by z-scores, representing how strongly each gene is predicted to be a member of each gene set. We used the implementation of DEPICT that is provided with the original publication [10].

#### 2.4.2 DIAMOnD

Previous research has described how genes associated with a disease are closely interconnected with each other in a protein-protein interaction network, referring to such groups of genes as “disease modules” [18]. Based upon this theory, the Disease Module Detection (DIAMOnD) algorithm has been developed by Ghiassian et al. [44]. DIAMOnD traverses a graph from a set of “seed” genes to identify additional genes of a module based upon their connectivity significance to the seed genes. Connectivity significance is determined by calculating the probability of the candidate being connected to seed genes and comparing that against the probability calculated under the null hypothesis. The authors compared the performance of their algorithm to random walks based upon 70 diseases and their associated disease genes. They found that the DIAMOnD algorithm was more suitable than random walks for identifying genes that are not in the immediate neighbourhood of the other genes in the module. Here, we test whether disease modules can be applied for our task with the DIAMOnD algorithm. For the leave-chromosome-out cross validation, we use DIAMOnD to predict genes on the left-out chromosome by using positive cases on the chromosomes that are used as training set as seed genes. Because DIAMOnD does not allow users to specify a set of genes to be ranked, we configured it to return the first 1000 genes for every chromosome that was used as a test set. From these 1000 genes, only the candidates of SNPs on the test chromosome were used, ranking them for each SNP based upon their position on the list as assigned by DIAMOnD.

#### 2.4.3 Network distance

Based on the theory that the disease genes associated with a disease are part of the same pathways or signal transduction mechanisms, Xu and Li defined five metrics that quantify the network distance between disease genes and candidate genes. These metrics were subsequently used as features to train and evaluate a supervised machine learning classifier to identify additional disease genes [38]. The five metrics are (1) the total number of neighbours of genes, (2) the fraction of neighbours which are disease genes in the training set, (3) the fraction of two-step neighbours which are disease genes in the training set, (4) the average network distance of the candidate gene to the disease genes in the training set, and (5) the average fraction of neighbours that a candidate has in common with the neighbours of the disease genes in the training set. The method achieved an average accuracy of 76% with cross validation using disease genes listed in OMIM as a reference [45]. Three newly predicted disease genes were validated in a literature search.

We implemented the five network-distance metrics with the Python library NetworkX [46].

#### 2.4.4 Graphlets

One theory about disease genes is that although they may not necessarily be near to each other in the network, they can be identified because their network topology is similar [39]. This network topology can be described by small-scale networks of which genes are part, referred to as graphlets [47]. These graphlets can be e.g. triangles, squares, pentagons, and can consist of up to five nodes. Identifying graphlets that consist of more than five nodes is uncommon because of the computational resources that would be required. Within the 29 graphlets that can be created with up to five nodes, there are 73 distinct positions (also referred to as orbits) a node can have. To count the number of orbits for each node, we use EVOKE, a recent, fast implementation of graph pattern counting methods [48].

Milenković et al. tested this theory by counting the number of orbits of each gene in the network, and used those frequencies to cluster the genes [39]. They predicted 31 new genes to be associated with cancer because these were part of clusters that consisted for at least 40% of known cancer genes. From these 31 predictions, they were able to validate 24 genes based upon the biomedical literature.

Graphlets were also previously combined with features generated with node2vec [35], as described in Section 2.4.5. We also report the results of the combination of these two methods. When combined with node2vec, the log of the frequencies of the graphlets was used as feature, which was not the case in the work of Milenković et al. Here we test both the original and the log-transformed frequencies as features.

#### 2.4.5 Node2vec

Node2vec is an unsupervised feature learning method which has gained in popularity in recent years [49]. Starting from every node in the graph, node2vec creates node embeddings by performing a user-specified number of biased random walks through the knowledge graph, which generate features that represent both the community (i.e. closely interconnected group of nodes) to which a node belongs, and its network role (e.g. whether the node is a hub). By not relying on pre-defined or rigid definitions of neighbourhood, node2vec is thought to describe nodes in a more flexible and comprehensive way as compared to e.g. graph statistics. As a result, each node in the graph is represented by a numerical vector, in which semantically similar entities are geometrically closer to each other.

Agrawal et al. tested features generated with node2vec stand-alone as well as combined with the log of the frequencies of graphlets as described in Section 2.4.4 [35]. They tested their methods on a set of 519 diseases and their associated disease genes as described in DisGeNet, and found that the inclusion of graphlets improved recall-at-100 from 0.300 to 0.332.

In other work, Peng et al. auto-encoded the features generated by node2vec prior to using them in a classifier [37]. They quantified the benefit of autoencoding by applying their method for the prediction of genes associated with Parkinson’s disease, showing an increase in performance from 67.0% AUC to 70.6%.

By default, node2vec is configured for undirected, unweighted networks. We modified these parameters to use the additional information available in our knowledge graph based upon the assumption that including more information improves performance. We therefore used the directional information of edges as well as the number of publications underlying an edge as edge weight. The Python implementation of node2vec was used to generate features.

We test the following variations of the features generated with node2vec (1) no modification, (2) adding the log of the frequencies of graphlets as described by Agrawal et al., (3) autoencoding the node2vec features as described by Peng et al., and (4) combining the autoencoding and the graphlets.

#### 2.4.6 RDF2vec

A limitation of node2vec is its inability to include predicate information in the feature generation process. To leverage the predicate information available in Resnet, we also tested RDF2vec, which extends upon node2vec by taking edge labels (predicates) into account when creating node embeddings. RDF2vec has not been used previously for disease gene identification.

Similar to node2vec, RDF2vec is an unsupervised feature learning method for knowledge graphs that maps the neighbourhood of a node by using the node as a starting point for random walks [36]. Features are generated based on the sequences of triples that result from these random walks. Contrary to node2vec, RDF2vec does not support weighted edges. To generate RDF2vec features we used pyRDF2vec as provided by VandeWiele et al. [50]. Similarly to node2vec, we tested whether autoencoding, enriching the features with the log of the frequencies of graphlets, or both, improved performance.

#### 2.4.7 Metapaths

Predicates between genes have not yet been used for disease gene identification, but previous research has leveraged predicates and their directional information from protein knowledge graphs to improve performance for drug efficacy screening and identification of disease trajectories [17, 19].

Here, we construct metapaths consisting of a maximum sequence of two predicates for every protein, taking into account directional information. Separate features are created for metapaths directed towards the protein (incoming metapath) and metapaths directed away from the protein (outgoing metapath). An example of a feature consisting of two predicates is “Incoming_metapath:Regulates-Expresses”. We test both binary features (i.e. absence or presence of metapaths) and numeric features (i.e. metapath frequencies).

## 3 Results

### 3.1 Extracted paths

We extracted 429,823 human protein–predicate–protein triples from Resnet. The triples contained 14,423 different proteins connected by 13 distinct predicates. The number of triples per predicate is shown in S5 Table.

### 3.2 Prediction results

**Fig 2:**
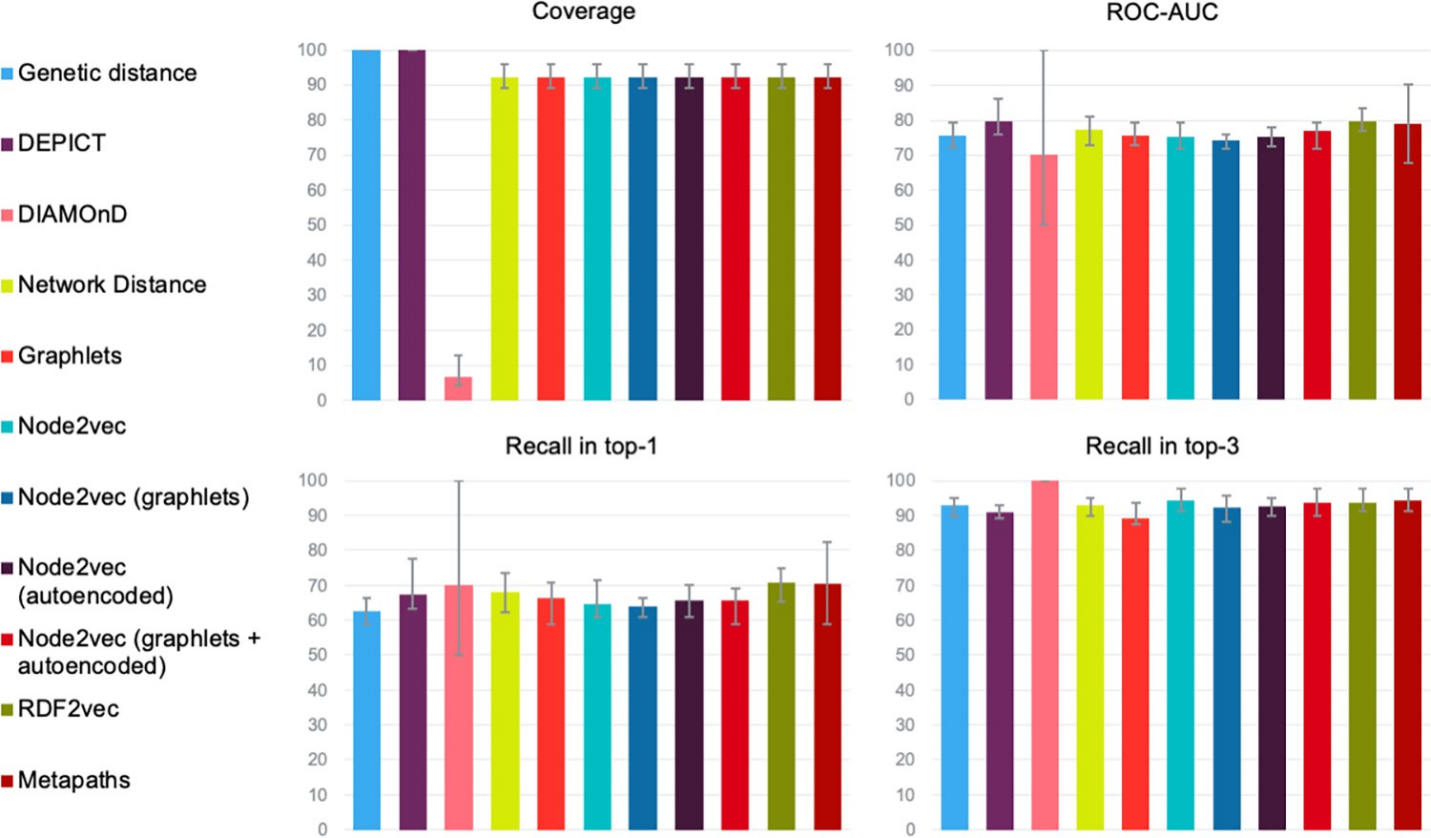
Average performance for different metrics, achieved by the best individual methods for identifying genes targeted by disease-associated non-coding SNPs. The x-axes list the different methods from left to right (the colours from left to right corresponding with those at the left listed from top to bottom), while the y-axes represent each of the four performance metrics as percentages ranging from 0% to 100%. Error bars indicate the range of the performances of the respective methods across the four reference sets.

The performance of the methods, in combination with their variations and classifiers that achieved the best result as determined by the highest AUC on average across all four reference sets, are shown in Fig 2 and Table 3. We only show the performance based on the candidates as found within the variable genomic intervals identified with DEPICT. Static intervals achieved considerably worse coverage or ranking performances. For example, coverage at a short interval of 25 kb was only 12%, while at the highest interval of 2000 kb none of the AUCs exceeded 60%. Performances of all individual reference sets, methods, variations, classifiers, and genetic intervals can be found in S6 Table.

**Table 3:** Performances achieved with different combinations of methods, variations, and classifiers. All values are percentages and indicate the average (minimum value–maximum value) across the four reference sets.

Method	Variation	Classifier	Coverage	AUC	Recall in top– 1	Recall in top– 3
**Genetic distance**			100	75.7 (72.1–79.3)	62.5 (58.7–66.2)	92.9 (89.7–95.1)
**DEPICT**			100	79.6 (75.9–86.2)	67.5 (63.3–77.5)	90.7 (89.1–93.0)
**DIAMOnD**			6.6 (4.1–12.7)	70.0 (50.0–100.0)	70.0 (50.0–100.0)	100.0 (100.0–100.0)
**Network Distance**		LR	92.1 (89.1–95.8)	77.3 (72.9–81.1)	68.0 (62.1–73.5)	92.9 (89.7–95.1)
**Graphlets**	log	LR	92.1 (89.1–95.8)	75.6 (73.0–79.3)	66.4 (58.7–70.6)	89.2 (87.4–93.5)
**Node2vec**	no modification	SVM	92.1 (89.1–95.8)	75.1 (71.9–79.5)	64.6 (61.0–71.3)	94.2 (91.3–97.6)
**Node2vec**	graphlets	LR	92.1 (89.1–95.8)	74.4 (71.8–75.8)	64.0 (60.9–66.2)	92.1 (88.2–95.7)
**Node2vec**	autoencoded	SVM	92.1 (89.1–95.8)	75.2 (72.4–78.1)	65.8 (60.9–70.1)	92.6 (89.7–95.1)
**Node2vec**	autoencoded, graphlets	SVM	92.1 (89.1–95.8)	77.0 (72.0–79.2)	65.5 (58.7–69.0)	93.5 (89.7–97.6)
**RDF2vec**	no modification	RF	92.1 (89.1–95.8)	79.6 (77.1–83.3)	70.6 (65.2–75.0)	93.7 (91.3–97.6)
**Metapaths**	binary	RF	92.1 (89.1–95.8)	78.9 (67.7–90.3)	70.5 (58.7–82.4)	94.1 (91.3–97.6)

Not all genes in the reference sets could be mapped to the protein–predicate–protein triples extracted from our knowledge graph (See S1 Table). Methods based on our protein knowledge graph covered on average 92.1% (min–max, 89.1–95.8%) of the positive cases found within the genetic intervals identified by DEPICT. DIAMOnD, which does not take the candidates within a genetic interval as input but has its own method to identify candidates, had a very low coverage of on average 6.6% (min–max, 4.1–12.8%) of the positive cases within the first 1000 candidates it identified. DEPICT only retrieved 46.9% of the positive cases as compared to taking a 2000 kb search window around the SNP (min–max, 25.7–73.2%). DEPICT managed to limit the average ratio of negative cases to positive cases to 2.1 to 1 (min–max, 1.8–2.3 to 1), compared to an average ratio of 44.2 to 1 at 2000 kb (min–max, 28.2–56.2 to 1).

When excluding DIAMOnD due to its low coverage (see upper left panel in Fig 2), methods that did not make use of the predicate information in the knowledge graph (i.e. node2vec, graphlets, and network distance) performed roughly equal to ranking genes based on their genetic distance from the SNP, which achieved an average AUC of 75.7% (see upper right panel Fig 2). Amongst these methods, only node2vec whose features were autoencoded and extended with graphlets, and network distance, achieved slightly higher average AUCs, of 77.0% and 77.3% respectively. Methods that included predicate information, i.e. RDF2vec and metapaths, performed best and achieved roughly similar ranking performance as DEPICT, which achieved an average AUC of 79.6%. The difference between methods with and without predicate information was 3.4 percentage points on average. To test whether the individual methods achieved significantly different ranking performances from each other we followed the procedure described by Demšar to compare multiple methods used on multiple datasets [51]. We applied the Friedman test to the AUCs that were achieved on the different reference sets and subsequently calculated the p-value according to Iman-Davenport. This showed that there were no statistically significant differences between the individual methods (*p* = 0.42). Recall in the top-1 ranged from 62.5% (genetic distance) to 70.6% (RDF2vec) on average, while recall in the top-3 ranged from 89.2% (graphlets) to 94.2% (node2vec) on average (see bottom panels Fig 2). While variations of node2vec achieved an approximately 5 percentage points lower recall in the top-1 than RDF2vec and metapaths, the recall in the top-3 was almost the same. However, the comparisons of ranking performances do not take into account the difference in coverage between the methods based upon protein knowledge graphs and DEPICT/genetic distances.

Autoencoding node2vec features, or extending them with graphlet counts, did not improve performance, nor did these variations improve performance when applied to RDF2vec. Only the combination of autoencoding and graphlets resulted in a 1.9 percentage point improvement for node2vec. Graphlets alone did benefit from using the log of the graphlet counts, and the metapaths benefitted from using binary features as compared to using their frequencies as features (see S4 Table for details).

The difference between the best and the worst classifier for the methods and variations was on average 5.3 percentage points AUC (min–max, 2.7–9.5). Three out of four variations of node2vec worked best with SVM, while the variation with graphlets worked best with logistic regression. Stand-alone graphlets and network distance performed best with logistic regression, while methods that incorporate predicate information (RDF2vec and metapaths) performed best with random forests. For half of the methods decision trees were the worst performing classifier, and for none of the methods the best. Amongst the four reference sets, performance achieved with the Teslovich reference set was best in 7 out of 10 experiments (DIAMOnD excluded), while performance achieved with the high-confidence Farashi set was lowest in half of the experiments.

### 3.3 Combining different methods

**Fig 3:**
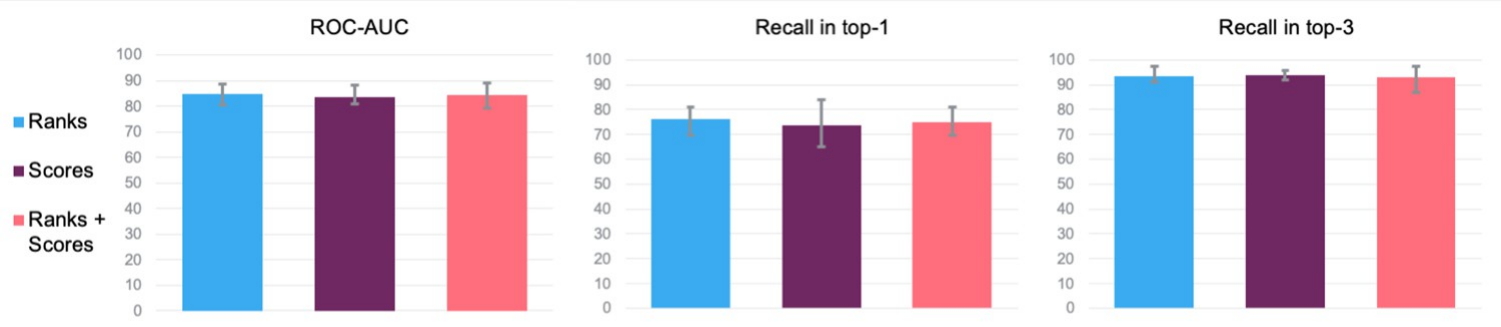
Average performance for different metrics, achieved by the best performing combinations of methods for identifying genes targeted by disease-associated non-coding SNPs. The x-axes list the different methods (represented by different colours), left to right corresponding with the datatype and best combination of methods in Table 4 from top to bottom. The y-axes represent each of the three performance metrics as percentages ranging from 0% to 100%. Error bars indicate the range of the performances of the respective methods across the four reference sets.

To investigate whether a combination of methods can improve performance, we trained new classifiers using the results (prediction scores and rankings of the gene candidates) of the methods listed in Table 3 as features. Due to its low coverage, the DIAMOnD algorithm was excluded from this experiment. We used logistic regression to train and evaluate new classifiers, applying the same leave-one-chromosome-out cross-validation procedure described in Section 2.3. The results are shown in Table 4 and Fig 3. The highest AUC (84.9%) was obtained for a combination of rankings from DEPICT, network distance, graphlets, node2vec, RDF2vec, and metapaths, improving performance with 5.3 percentage points as compared to RDF2vec and DEPICT, the best single methods. Testing the difference in performance of all ten individual methods along with the three combined methods with the Friedman test, again calculating the p-value with the Iman-Davenport formula, showed that their difference was significant (*p* = 0.0002).

**Table 4:** Best performances of combinations of methods across the four reference sets. All values are percentages and indicate the average (minimum value–maximum value).

Data type	Methods included	AUC	Recall in top 1	Recall in top 3
Ranks	DEPICT, Network distance, Graphlets, Node2vec, Metapaths, RDF2vec	84.9 (80.3–88.7)	76.1 (69.6–80.9)	93.5 (91.3–97.6)
Scores	DEPICT, Graphlets, Node2vec, Node2vec + graphlets, Metapaths	83.4 (80.7–88.2)	73.7 (65.2–83.8)	94.0 (91.9–95.6)
Scores & Ranks	DEPICT, Graphlets, Node2vec, RDF2vec, Metapaths	84.5 (79.4–88.8)	74.9 (69.6–80.9)	92.9 (87.0–97.6)

When testing the difference in performance between the individual methods that were used together in the combined method for ranks (DEPICT, Network distance, Node2vec, RDF2vec, metapaths), and their combined method itself with the Friedman test, the difference was significant (*p* = 0.04). We again followed Demšar for our post-hoc analysis by performing the Dunn-test and using Holm’s step-down procedure to correct for multiple testing [51]. This showed that the combined method based on ranks differed significantly with the graphlets (*p* = 0.006) and node2vec (*p* = 0.01) methods. Using both prediction scores and rankings improved the AUC as well, but somewhat less (84.5%). Here, the Friedman test did not result in a significant p-value when testing the difference in performance between the combined method that used both scores and ranks, and the individual methods that were part of it (DEPICT, graphlets, node2vec, metapaths, rdf2vec) (*p* = 0.076). Combining only prediction scores improved the AUC least, achieving an average AUC of 83.4%. However, the difference between the individual methods that were used in the combined method for scores (DEPICT, graphlets, node2vec, node2vec + graphlets, metapaths) and the combined method for scores itself was significant (*p* = 0.03). The Dunn post-hoc analysis, corrected with Holm’s step-down procedure, showed that the difference was significant between the combination of methods for scores and node2vec + graphlets (*p* = 0.006).

## 4 Discussion

Identification of the genes being targeted by SNPs that are located on non-coding parts of the genome remains challenging. Here, we explored whether a protein knowledge graph can be used to identify these genes by comparing the performances of six existing methods, four of which have previously been developed for disease gene identification, with two baselines. Performances of all tested methods averaged over four reference sets varied between 70% and 80% AUC, suggesting that the roles that genes play within a protein knowledge graph can be used to identify genes that are targeted by non-coding SNPs. Amongst the methods that we tested we find that RDF2vec and DEPICT achieved the highest average AUC, followed by metapaths. Methods based on the protein knowledge graph that used predicate information, i.e. RDF2vec and metapaths, both performed better than methods that did not, i.e. node2vec, graphlets, and network distance, demonstrating the added value of using predicate information for our task. Prioritization based on genetic distance, one of our baselines and the leading assumption amongst geneticists, performed roughly equal to methods lacking predicate information. Yet while the above trend was clear based on the AUCs, our four reference sets did not provide sufficient power for the differences to be significant when tested with a Friedman test. Furthermore, because some proteins from our reference sets were disconnected from other proteins in our knowledge graph, these were excluded from further analysis. Genetic distance and DEPICT did not share this dependence on our protein knowledge graph, leading to differences in coverage between methods, which complicated the direct comparison of their performances.

Combining methods improved performance up to 5.3 percentage points AUC. When testing the difference between the combined methods for ranks and scores to the individual methods which were included in them, statistically significant differences were found. This was not the case when using both ranks and scores simultaneously. Our best performing combinations of methods all included DEPICT as well as at least one of the two methods that used predicate information. Therefore, although none of the methods based on a protein knowledge graph outperformed DEPICT, the results of our combination experiments show that combining DEPICT with (predicate) features from a protein knowledge graph improves performance. Genetic distance, the leading assumption amongst geneticists and our other baseline method, was not included in the best performing combinations of methods.

While previous authors have reviewed network-based methods for prioritizing candidate genes for SNPs [52, 53], to our knowledge we are the first to systematically compare their performance by applying them to the same reference sets, and comparing them to baselines. Furthermore, we compared different ways to determine the search intervals around SNPs for gene candidates. In addition to the commonly used AUC we also evaluated our performance by calculating the recall in the top-1 and top-3 results, which users may find more straightforward to interpret and practically relevant. Given the large number of published methods to extract features from knowledge graphs, it is infeasible to test them all. In this study we limited ourselves to testing existing disease gene identification methods that use protein-knowledge graphs. Future research may include different methods to extract features from knowledge graphs, beyond those that have specifically been used for disease gene identification. Also, we only tested our methods on a single knowledge graph, which is based on triples extracted from the biomedical literature, while Franke et al. have demonstrated that combining different types of sources improves performance [54]. Contrary to previous studies, our knowledge graph contained predicates between proteins, enabling us to test and demonstrate their added value.

Directly comparing our results to those achieved by the original disease gene identification studies is complicated because of the difference in objectives. While we identified gene candidates for individual SNPs, previous methods identified gene candidates across the whole genome or for a locus. A comparison to DEPICT is complicated because its developers tested their method on only the LDL subset of the Teslovich reference set, for which they achieved a perfect prediction performance.

Contrary to previous research in which autoencoding features generated by node2vec improved performance for disease gene identification [37], autoencoding node2vec features failed to improve performance for our task. Similarly, extending node2vec with frequencies of graphlets also did not improve performance [35]. Only the combination of autoencoding and graphlets improved performance by 1.9 percentage points AUC. The combination of node2vec and the graphlets did improve performance when combined with other methods. For the RDF2vec features, applying the same modifications failed to improve performance in general.

We compared different static intervals around a SNP to identify candidate genes, in addition to the intervals around SNPs based on linkage disequilibrium. We found that static intervals result in either poor coverage or poor ranking performance. While short intervals such as 25 kb achieved a coverage of 12% on average, at higher intervals of 2000 kb none of the AUCs exceeded 60%. In contrast, DEPICT’s linkage-disequilibrium-based method results in both a relatively high coverage and one of the best ranking performances. Yet this method also has drawbacks. DEPICT determines the interval around a SNP based on the HapMap data and data from the 1000-genomes project, which may be biased towards certain populations or diseases. Furthermore, DEPICT is unable to identify intervals around SNPs on the X-chromosome (which means that none of the presented results apply to the X-chromosome). The output of DEPICT does not offer an explanation why for some SNPs no candidates are found. On average, DEPICT retrieved less than half of the positive cases from our reference sets. Future research could investigate whether lowering the current r^2^ cutoff of 0.5, which DEPICT uses to determine the genetic intervals around SNPs, would improve coverage, while maintaining ranking performance. Alternatively, using tools other than DEPICT to identify the gene candidates for SNPs could be explored. For example, FUMA [6] is a commonly used tool for post-GWAS analyses which also accepts GWAS summary statistics as input, while gene candidates for SNPs identified by the OpenTargets algorithm [11] have been made available in a publicly accessible database.

Because the disease gene identification methods that we tested use protein-protein interaction networks, we limited our task to protein-coding genes. SNPs targeting other coding sequences, such as long non-coding RNAs or pseudogenes were excluded. Each reference set contained different numbers of non-coding sequences. None were found in the Teslovich set, 16 in the full Farashi set, and 69 for the DeRycke reference set (24 of these were subunits of the *HLA* and *RP11* genes). Some of these non-coding sequences are entities within Resnet. Future research may examine if features derived from a protein knowledge graph for such non-coding sequences could be used in a similar way as those of protein-coding sequences to determine whether they are targeted by SNPs.

Although our graph-based methods use supervised learning in contrast to DEPICT’s unsupervised gene-set based approach, it might be worthwhile to investigate whether unsupervised learning methods achieve similar performance. For example, Milenković et al. clustered genes together based upon their orbit frequencies within graphlets, after which they evaluated whether disease genes were clustered together [39]. A similar methodology could be tested for our task. If genes targeted by non-coding SNPs can indeed be clustered together with high accuracy based on features derived from a protein knowledge graph, that would suggest that these genes have common network properties which are independent from a specific disease or training set. A precedent for such common network properties of genes associated with disease is the lethality-centrality theory, which poses that network connectivity of disease genes is relatively high, but not high enough to be fatal [55].

Our work demonstrates that methods for protein knowledge graphs can be used to identify genes that are targeted by SNPs located on the non-coding part of the genome. By achieving two out of the top-three performances across all four reference sets, we show the added value of predicate information within these knowledge graphs. Combining methods improved performance even further, up to 84.9% AUC on average. However, in practice users may also weigh aspects other than performance, such as comprehensibility and ease of implementation in selecting a method. Our results show that the current leading assumption amongst geneticists, that a SNP targets the nearest gene, remains a valid choice for users who are willing to accept slightly lower performance in favour of comprehensibility and ease of implementation. This method only requires the locations of the SNP and genes on the genome, which is information which is easily available, but achieves a 3.9 percentage point lower AUC than the best individual methods, DEPICT or RDF2vec, and already finds 92.9% of the positive cases in the top-3 results. Network distance offers a slightly better performance (1.6 percentage points AUC on average) as compared to genetic distance, whilst its five features are comprehensible and relatively easy to implement. Users who prefer the highest performance would best use a combination of methods, thereby gaining 9.2 percentage points AUC over genetic distance. Alternatively, users may consider using methods in sequence, starting with genetic distance, trying network distance for the SNPs for which genetic distance fails to yield plausible results, and continuing to use increasingly complex methods for those SNPs for which only implausible candidates are highly ranked. Although such a sequence of methods may entail more work than using a single method, it may offer an attractive compromise between ease of use, comprehensibility, and overall performance.

## Supporting information

S1 Table Number of genes and SNPs excluded from the experiments. (XLSX)

S2 Table Farashi reference set. Columns indicate the rs identifier of the SNP (SNP ID), the chromosome on which the SNP is found and its location (chromosome & location), the p-value as taken from Farashi’s overview (GWAS/eQTL p-value), the ENSEMBL identifier of the gene candidate (ENSEMBL ID), the gene symbol (gene symbol), the class of the SNP-gene pair, indicating whether it is a positive case or a negative (Class), the base pair distance as a positive integer between the SNP and the start/end of the gene (whichever value is lower) (base pair distance absolute), the base pair distance between the SNP and the start/end of the gene (whichever value is lower), where negative values indicate the gene is located upstream from the SNP (base pair distance), and the gene rank as determined by the absolute base pair distance (Gene rank).
(CSV)

S3 Table DeRycke reference set. Columns indicate the rs identifier of the SNP (SNP ID), the chromosome on which the SNP is found and its location (chromosome & location), the ENSEMBL identifier of the gene candidate (ENSEMBL ID), the gene symbol (gene symbol), the class of the SNP-gene pair, indicating whether it is a positive case or a negative (Class), the base pair distance as a positive integer between the SNP and the start/end of the gene (whichever value is lower) (base pair distance absolute), the base pair distance between the SNP and the start/end of the gene (whichever value is lower), where negative values indicate the gene is located upstream from the SNP (base pair distance), and the gene rank as determined by the absolute base pair distance (Gene rank).
(CSV)

S4 Table Teslovich reference set. Columns indicate the rs identifier of the SNP (SNP ID), the chromosome on which the SNP is found and its location (chromosome & location), the p-value as taken from the meta-analysis by Teslovich (p-value), the ENSEMBL identifier of the gene candidate (ENSEMBL ID), the gene symbol (gene symbol), the class of the SNP-gene pair, indicating whether it is a positive case or a negative (Class), the base pair distance as a positive integer between the SNP and the start/end of the gene (whichever value is lower) (base pair distance absolute), the base pair distance between the SNP and the start/end of the gene (whichever value is lower), where negative values indicate the gene is located upstream from the SNP (base pair distance), and the gene rank as determined by the absolute base pair distance (Gene rank).
(CSV)

S5 Table Predicates and their frequencies as found in the human protein–protein triples extracted from Resnet. (XLSX)

S6 Table Prediction performances of all methods, variations, and classifiers, at all base pair distances, for all reference sets. Columns indicate the reference set to which the method, variation, and classifier were applied, and the performance that was achieved by them. AUC indicates the average AUC across all SNPs in the reference sets. Hits@1/3/5/10 indicate the absolute number of results that were returned in the top 1/3/5/10. The recall can be calculated by dividing these by the total number of positive cases that could be found within the selected genetic range around the SNP. Mean rank and Median rank indicate the mean rank and median rank of positive cases respectively.
(CSV)
